# Human embryo research, stem cell-derived embryo models and *in vitro* gametogenesis: Considerations leading to the revised ISSCR guidelines

**DOI:** 10.1016/j.stemcr.2021.05.008

**Published:** 2021-05-27

**Authors:** Amander T. Clark, Ali Brivanlou, Jianping Fu, Kazuto Kato, Debra Mathews, Kathy K. Niakan, Nicolas Rivron, Mitinori Saitou, Azim Surani, Fuchou Tang, Janet Rossant

**Affiliations:** 1 University of California, Los Angeles, CA, USA; 2 The Rockefeller University, New York, NY, USA; 3 The University of Michigan, An Arbor, MI, USA; 4 Osaka University, Yamadaoka, Osaka, Japan; 5 Johns Hopkins University, Baltimore, MD, USA; 6 Francis Crick Institute and The Centre for Trophoblast Research, University of Cambridge, Cambridge, UK; 7 Institute of Molecular Biotechnology of the Austrian Academy of Sciences, Vienna BioCenter, Vienna, Austria; 8 Kyoto University, Sakyo-ku, Kyoto, Japan; 9 The Gurdon Institute, Cambridge, UK; 10 Beijing Advanced Innovation Center for Genomics, Beijing, China; 11 Gairdner Foundation, Toronto, Ontario, Canada

**Keywords:** embryo research, embryo models, guidelines, gametoogensis

## Abstract

The ISSCR Guidelines for Stem Cell Research and Clinical Translation were last revised in 2016. Since then, rapid progress has been made in research areas related to *in vitro* culture of human embryos, creation of stem cell-based embryo models, and *in vitro* gametogenesis. Therefore, a working group of international experts was convened to review the oversight process and provide an update to the guidelines. This report captures the discussion and summarizes the major recommendations made by this working group, with a specific emphasis on updating the categories of review and engagement with the specialized scientific and ethical oversight process.

## Teaser

In this perspective, Rossant, Clark, and colleagues outline the process and deliberations that led to the updated ISSCR Guidelines for stem cell research involving the 14-Day rule, stem cell-based embryo models, and *in vitro* gametogenesis.

## Main text

### Framing the issues

The ISSCR Guidelines for Stem Cell Research and Clinical Translation were last revised in 2016. At that time, it was already recognized that the ethical issues related to human embryo research extended well beyond the use of human embryos for generation of embryonic stem cells (ESCs). The 2016 guidelines considered broader issues related to human embryo research, including generation of embryos specifically for research, *in vitro* culture of human embryos, stem cell-embryo chimeras, and genome editing of human embryos. The 2016 guidelines also proposed that all research related to human embryos be subject to oversight by a special process, named Embryo Research Oversight (EMRO), and provided guidance on proposed categories of research that could be allowed, reviewed, or prohibited under such a process.

Since 2016 there has been rapid progress in several areas of human embryo-related research, including technologies for extended *in vitro* culture of human embryos up to 14 days, creation of stem cell-based embryo models that reflect different stages of human embryo development, and *in vitro* gametogenesis (IVG) from stem cells. In the light of the changing science, there was a need to revisit the oversight process and the categories of research to be reviewed. A sub-committee of the Task Force to update the ISSCR Guidelines called Working Group 2, was specifically charged with reviewing this area and proposing appropriate revisions to the guidelines. The working group was chaired by Amander Clark and Janet Rossant, and included scientists with relevant expertise and ethicists involved in stem cell/embryo oversight issues (please refer to the author list). The working group had extensive discussions and debates over a 14-month period. Subgroups within this working group focused on particular areas but in the end the entire working group agreed by consensus on the proposed recommendations. These were then further reviewed by the full Guidelines Task Force, the Board of ISSCR, the ISSCR Ethics Committee, and an invited group of regulatory and ethics experts before being subject to external peer review. The final guidelines were approved by the ISSCR Board in December 2020. Guidelines on related research, including germline genome editing and chimera formation were not the purview of Working Group 2, and the deliberative process from these groups are not included here. A white paper on the issues associated with creating chimeric embryos using human stem cells, or contribution of human cells to the germline of chimeras can be found in Hyun et al. (2020).

### General framework

Our working group’s task was to review the existing guidelines and recommend additions and/or modifications to account for the changing science and societal issues. We accepted the general principles underlying the oversight and review process as outlined in the 2016 guidelines, as well as the general concept of a specialized process for review of human embryo- and stem cell-related research. The name “EMRO” was removed from the updated 2021 guidelines recognizing that the specifics of the oversight process would vary in different jurisdictions. Working Group 2 focused on the proposed review categories and the types of research that should fall under each heading.

The 2016 guidelines had three categories of review; the new guidelines divide two of these categories, to provide clearer delineation of the different levels of review.

**2016 Category 1:** Exempt from review
  **New Category 1A:** Exempt from review
  **New Category 1B:** Reportable to an oversight process but normally exempt from review
**2016 Category 2:** Requires review; category is unchanged although the areas of research under this heading have increased
**2016 Category 3:** Prohibited research activities
  **New Category 3A:** Research activities currently not permitted
  **New Category 3B:** Prohibited research activities

The rationale for these changes, as well as the areas of research that would fall into the different categories are described in more detail in the following sections. It is important to note up front that the culture of human embryos or organized embryo-like structures beyond 14 days, or formation of the primitive streak, whichever occurs first (herein referred to as the “14-day rule”), has been removed from category 3, prohibited activities. This was the subject of many levels of discussion, debate, and consultation over many months. While recognizing that human embryo culture beyond 14 days is prohibited by law or regulation in many jurisdictions, the committee felt that this is an area where a blanket prohibition could inhibit important research directions. The scientific, ethical, and regulatory background to this recommendation is discussed further in later sections.

#### The decision to update the categories of scientific and ethical review

The decision of the committee to update the laboratory science covered by the different categories reflects not only the changing landscape of stem cell research but also the challenge in defining the concept of “organismal potential,” which was previously proposed as a parameter in reviewing research activities in category 2 or assigning the research to category 3. Furthermore, the committee considered the concept of “time” as part of the 14-day rule to be of limited value when considering the new stem cell-based embryo models given that fertilization is not the starting point to generate a model of the human embryo.

In the case of human stem cell-based embryo models, rather than “organismal potential” we instead proposed a grading of ethical and scientific oversight based on the degree of integration. This is because some embryo models mimic only specific aspects/tissues of human embryo development (non-integrated models), whereas others are designed to model the integrated development of the entire early human conceptus. The models in the first category do not have any reasonable expectations of specifying additional cell types that would result in formation of an integrated embryo model. In contrast, models in the second category might manifest the ability to undergo further integrated development when cultured for additional time *in vitro*. Therefore, the more integrated the model, the higher the ethical oversight. The committee updated the glossary to define the concepts of an integrated versus non-integrated model of human embryo development.

Based on these discussions, some examples of research activities that should now be considered under the updated categories of review are as follows. For additional examples, please refer to the updated guidelines (ISSCR.org/guidelines).

**Category 1A:** Research that is permissible after review under existing mandates and/or committees and determined to be exempt from the specialized oversight process. For example:
  a Research with human pluripotent stem cell lines that is confined to cell culture and/or involve routine research practices, such as assays of *in vitro* differentiation and teratoma formation.
**Category 1B:** Research that is reportable to the oversight process but not normally subject to further review, at the discretion of the appropriate committee and/or local policy. Some examples include:
  a Research that entails the *in vitro* formation of human stem cell-based embryo models that are not intended to represent the integrated development of the entire embryo.
  b Research on *IVG* from cells, including genetically modified pluripotent stem cells, which does not involve attempts at fertilization and the generation of embryos.
**Category 2:** Forms of research with embryos and embryo models that are permissible only after review and approval through a specialized scientific and ethics review process. Some examples include:
  a Research involving the *in vitro* culture of human embryos where embryos are maintained in culture until the formation of the primitive streak or 14 days, whichever occurs first.
  b Generation of stem cell-based embryo models that represent the integrated development of the entire embryo, including its extra-embryonic membranes. These integrated stem cell-based embryo models should be maintained in culture for the minimum time necessary to achieve the scientific objective.
  c Research that generates human gametes from any progenitor cell type *in vitro*, when this entails performing studies of fertilization that produce human zygotes and embryos.
**Category 3A:** Research activities currently not permitted. Research under this category should not be pursued at this time because the approaches are currently unsafe or raise unresolved ethical issues. Some examples include:
  a The use of human gametes differentiated from human stem cells for the purposes of fertilization and human reproduction.
  b Research in which human embryos that have undergone modification of their nuclear genome are transferred into or gestated in a human uterus.
**Category 3B:** Prohibited research activities. Research under this category should not be pursued because of broad international consensus that such experiments lack a compelling scientific rationale and are widely considered to be unethical. Such research includes:
  a Transfer of human stem cell-based embryo models to the uterus of either a human or animal host.
  b Research in which animal chimeras incorporating human cells with the potential to form human gametes are bred to each other.
  c Transfer of a human embryo(s), irrespective of its origins, to an animal uterus.

In addition to the expanded interest and activity of research using stem cell-based embryo models, the committee also recognized that significant progress has been made with the differentiation of human stem cells and germ cells toward IVG. In the updated guidelines, the committee proposes that IVG should be subject to category 1A and category 1B. However, the formation of embryos after fertilization (or parthenogenesis) of IVG-derived gametes, should require full review under category 2. The use of human gametes differentiated from stem cells for the purposes of human reproduction currently falls under a prohibited activity, category 3A, as the committee decided that safety issues around this technology remain to be resolved. In the following sections, the committee’s deliberations around human embryo models, working with human embryos in culture and the relevance of the 14-day rule, as well as IVG will be discussed.

### Human embryo models

#### Terminology of embryo models

Over the last few years, human pluripotent stem cells cultured *in vitro* have demonstrated a capacity to spontaneously organize into structures resembling aspects of the developing early embryo. Because these human embryo models can be formed in large numbers and modified either genetically or physically with greater versatility as compared with human embryos, they represent powerful *in vitro* assays to understand human embryogenesis and early pregnancy loss. These embryo models do not arise from fertilization or nuclear transfer, they mimic a short developmental window (typically a few days), and in some cases only mimic specific aspects/tissues of human embryo development. As such, stem cell-based embryo models should not be considered equivalent to human embryos under most legislation (Table 1). Considering the proportionality (balancing the benefits and harms) and subsidiarity (pursuing goals using the morally least problematic means) of human embryo research, the committee recognized that embryo models are an ethical alternative to the use of embryos for *in vitro* research. The revised guidelines have incorporated these embryo models under existing ethical frameworks so as to ensure that research advances in agreement with ethical and societal goals (Pereira Daoud et al., 2020; Hyun et al., 2020; Rivron et al., 2018a; Sawai et al., 2020).

**Table 1:** Definitions of embryos and human stem cell-based embryo models, including categories under which each embryo type are reviewed

Embryo type	Definition	Category of review
Human embryo	formed by fertilization of a human oocyte by a human sperm, including an oocyte and/or sperm generated by IVG	category 2
Parthenogenetic human embryo	formed without the contribution of human sperm	category 2
Nuclear transfer human embryo	formed by the enucleation of the human oocyte and replacement of the nuclear genome by nuclear transfer	category 2 for *in vitro*, category 3B for *in vivo* gestation
Integrated stem cell-based human embryo model	contain the relevant embryonic and extra-embryonic cell types and could potentially achieve sufficient complexity to undergo further integrated development	category 2
Non-integrated stem cell-based human embryo model	mimic specific aspects/tissues of human embryo development	category 1B
Chimeric embryo (not considered a human embryo)	formed by transferring human cells into a non-human embryo followed by culture *in vitro*	category 1B

In addition, to best reflect the state and the envisioned applications of these structures made from stem cells, the use of the umbrella term “embryo model” or “stem cell-based embryo model” is encouraged, while the use of the term “synthetic embryo” or “artificial embryo” or “embryoids” should be avoided. Furthermore, the establishment of a terminology precisely reflecting the degree of integration and the type of model is encouraged (e.g., *post-implantation amniotic sac embryoid [PACE]* [Zheng et al., 2019], *blastoid* [Rivron et al., 2018a]).

#### Integrated versus non-integrated embryo models

Here, we propose a classification of human embryo models with the aim of guiding the decisions of the scientific and ethical oversight process. The *non-integrated embryo models* will be models that mimic only specific aspects/tissues of human embryo development and often do not have any associated extra-embryonic membranes. *These non-integrated embryo models* are reportable but not normally subject to further review (category 1B). In contrast, the *integrated embryo models* which contain the relevant embryonic and extra-embryonic cell types and could potentially achieve the complexity where they might realistically manifest the ability to undergo further integrated development if cultured for additional time *in vitro* should be subjected to a full specialized review (category 2). Given that the stem cell-based embryo models are not considered equivalent to human embryos under most legislation (as described in detail above), the decision was made that the integrated embryo models should not be subject to the restrictions of the 14-day rule. In addition, for both ethical and safety reasons, transferring any human embryo model into the uterus of a living animal or human is prohibited (category 3B).

#### Examples and potential applications

By recapitulating *in vitro* early human embryonic events, the use of human embryo models for scientific discovery opens ethical alternatives to addressing important biomedical problems. For example, in the next decade, *non-integrated human embryo models* are likely to model specific events that occur during the first few months of human embryo development, including gastrulation, body axis formation, and somitogenesis, thus allowing the investigation of numerous aspects of embryogenesis-related pregnancy problems and genetically inherited defects. Furthermore, the *non-integrated embryo models* are likely to help researchers to gain basic knowledge of the specific molecular and cellular events associated with genome mutations associated with developmental origins of disease. They should also guide drug discovery and biomedical strategies aiming at managing genetic diseases or forming or regenerating complex organs for regenerative medicine. Examples of such models include human pluripotent stem cells grown on micropatterned two-dimensional surfaces with confined geometry (Warmflash et al., 2014), *gastruloids* (Moris et al., 2020; van den Brink et al., 2014), *PACE* (Zheng et al., 2019), or *neuruloids* (Haremaki et al., 2019).

Stem cell-derived *blastoids* that mimic the blastocyst stage of development have been produced in the mouse (Kime et al., 2019; Li et al., 2019; Rivron et al., 2018b; Sozen et al., 2019) and very recently in the human (Liu et al., 2021; Yanagida et al., 2021; Yu et al., 2021). In the next decade, such *integrated human embryo models* are likely to progress from the blastocyst equivalent stage through the steps of early post-implantation development, including human primitive streak formation, gastrulation, formation of the embryonic germlayers, and specification of primordial germ cells (PGCs) thus allowing the study of numerous processes that require interactions between the embryonic and extra-embryonic tissues*. Integrated embryo models* are likely to guide drug discovery and biomedical strategies aiming at managing early pregnancy to address global health issues, such as infertility as a consequence of unexplained early pregnancy loss, development of new non-hormonal contraception technologies, or formulation of new culture conditions that could be used to improve *in vitro* fertilization (IVF) culture media.

### Working with human embryos in culture, and the relevance of the 14-day rule today

#### Deliberation process

Recent technological advances now allow *in vitro* culture of human embryos for up to 14 days (Deglincerti et al., 2016; Shahbazi et al., 2016). These studies and others that followed have unveiled some molecular and cellular events that occur at post-attachment stages of human embryonic development, the discovery of species-specific attributes of early embryo development and have highlighted the limitation of using model organisms in extrapolating information to human embryogenesis (Gerri et al., 2020). For example, the existence of a species-specific yolk sac trophectoderm tissue in human embryos could not have been extrapolated from model systems, such as the mouse (Deglincerti et al., 2016). Similarly, while studies in non-human primates show close comparators with human development (Nakamura et al., 2017; Sasaki et al., 2016), equivalence to humans should not be assumed. It has been reported that non-human primate embryos have been successfully cultured up to 21 days, including through the gastrulation period (Ma et al., 2019; Niu et al., 2019), suggesting that it should be technically feasible to successfully culture human embryos beyond 14 days.

The 14-day rule has been a broadly adopted limit on the culture of human embryos (Matthews and Morali 2020). This “rule” is in many cases not legally binding and instead is an intended acknowledgment of, and compromise with, the range of strongly and deeply held beliefs about the moral status of human embryos across some, but not all cultures and religions—an effort to allow some scientifically valuable research to move forward, within societally agreed limits (Hug, 2006; Hyun et al., 2016; Warnock, 1984; Williams and Johnson, 2020). Of note, going beyond the 14-day limit never became an active issue until recently, because human embryos could not be kept alive in culture beyond about a week. While the 14-day rule was somewhat arbitrary, it does define a clear developmental window before the body axis and the nervous system begin to form and after which twinning is no longer possible.

Given the technical advances described above, some jurisdictions have begun to reconsider the 14-day rule, motivating the panel to engage in an extensive deliberation about the potential benefits and risks of extending the 14-day rule. The panel was predominantly in favor of extending the redline, although dissenting opinions were also voiced. All of the group ultimately agreed to remove “culture of human embryos beyond 14 days or primitive streak formation” from the category of prohibited activity under category 3. Since the ISSCR Guidelines are only re-evaluated every 5 years, it was felt that now was the time for the community to engage in meaningful and substantial public communication and deliberations. Given advancements in human embryo culture, and the potential for such research to yield beneficial knowledge that promotes human health and wellbeing, national academies of sciences, academic societies, funders, and regulators should lead public conversations on the scientific significance as well as the societal, moral, and ethical issues of allowing such research. It should not be assumed that the public will necessarily support the extension of the 14-day rule, which was historically an important policy position fostering public trust in research and acknowledging broadly held social values. If such conversations do lead to broad public support for the research within a jurisdiction, and if local policies and regulations permit, embryo culture beyond 14 days and into primitive streak formation and gastrulation could be considered in those jurisdictions for review by the specialized oversight process under category 2. Such a review should carefully consider whether the scientific objective of the research justifies the time in culture beyond 14 days and ensure that only a minimal number of human embryos are used to achieve the research objectives. Established ISSCR Guidelines for research projects aimed at illuminating the events up to 14 days post fertilization or before primitive streak formation will remain the same.

To aid in the ongoing debate, we provide some of the considerations that were aired in the committee deliberations on the 14-day rule. The arguments in favor of maintaining the 14-day rule for the time being are that the second week of embryonic development has only recently become accessible for study, and there is still much to be learned between 7 and 14 days post fertilization. In addition, the scientific community should demonstrate for the public the value of the original compromise—What has been learned about the first 7 days of human development? and What impact has the knowledge made on clinical care? The scientific community needs to take the time to justify for the public revisiting the previously agreed compromise. Furthermore, the methodologies for culturing human embryos up to 14 days have recently been developed and may require further optimization, for example to consistently maintain a yolk sac cavity. Arguments in favor of extending the limit were largely based on the potential scientific and clinical benefits. There is a considerable gap of knowledge between the first 2 weeks of human development and the fourth week of life; a time that involves high rates of early pregnancy loss, thus making this stage very challenging yet extremely important to study. Preclinical assessment of this developmental stage would be particularly informative for future advances in mitochondrial replacement therapy, IVG, or germline genome editing. There is an increasing need to perform comparative studies of human embryos to stem cell-derived embryo models, allowing for the assessment of the fidelity of *in vitro* stem cell-based embryo model systems. If validated, these embryo model systems can be used in the future instead of human embryos to study the cell and molecular events that occur during and after primitive streak formation. There are also several direct clinical implications to studying human embryos beyond the 14-day rule. Early congenital diseases, and some late-onset diseases (Gluckman et al., 2008), have their roots in early embryogenesis. Examples include autism (Miller et al., 2005), heart malformation (Anderson et al., 1974), and neural tube defects (Greene and Copp, 2014). Advances in our understanding of such diseases would require a knowledge of the cellular and molecular events that occur during the development of the nervous system, the heart, and other organs, which would require extending the limits on *in vitro* culture close to Carnegie stage 12 (day 26–30). In addition, *in vitro* culture of human embryos would decrease the burden on the experimental use of animals, especially non-human primates. Individuals who donate human preimplantation embryos to research do so following informed consent with counseling available. Donation is nearly uniformly of material that is surplus to IVF treatment, which would be otherwise destroyed, and is often viewed by the donating individual as positively contributing to future clinical improvements.

In summary, the future of human embryo culture beyond 14 days to study gastrulation and post-gastrulation events, such as primitive streak formation, early germ layer development, formation of PGCs, and early organogenesis remains to be determined and will certainly run into different barriers in different jurisdictions. In several countries there is a legal ban on human embryo culture beyond 14 days and there are regulatory restrictions in many others (Matthews and Morali, 2020).

### Human IVG—Where to draw the regulatory line today

Generation of gametes *in vitro* (IVG) from human cells provides the opportunity to study human germ cell development, including the processes of imprint erasure, imprint resetting, and meiosis. Failure to erase and reset imprints can lead to the birth of children with developmental disabilities. Furthermore, aneuploidies arising through meiotic errors can lead to either pregnancy loss or children born with chromosomal conditions leading to morbidity and mortality. Therefore, understanding the process of human germ cell development, including the mechanisms of imprinting and meiosis, are essential to understanding infertility and diseases that impact human reproduction and child health. In this section, the promise of IVG will be highlighted based on work using the mouse. This will be followed by oversight and review considerations for performing IVG with human cells.

The committee’s deliberations on IVG focused on the use of human ESCs (hESCs) and human induced pluripotent stem cells (iPSCs) given the recent success using mouse cells (Hayashi et al., 2011, 2012; Ohinata et al., 2009; Hikabe et al., 2016; Zhou et al., 2016). Specifically, IVG with mouse ESCs or iPSCs involves first differentiation into epiblast-like cells followed by a second step of differentiation into primordial germ cell-like cells (PGCLCs). PGCLCs are diploid germ cells with the potential to differentiate into oogonia-like cells (Hikabe et al., 2016) or male germline stem cell-like cells (GSCLCs) (Ishikura et al., 2016) that undergo meiosis to become gametes (oocytes or sperm) when cultured or transplanted into an appropriate niche. Notably IVG to create fertilization-competent oocytes requires a final step of *in vitro* maturation (IVM) before successful fertilization and the birth of healthy offspring (Hikabe et al., 2016). In addition to starting with ESCs and iPSCs, gametes have also been created *in vitro* from mouse organ cultures using prenatal ovaries (Morohaku et al., 2016); primordial follicle culture followed by IVM (Eppig and O'Brien, 1996; O'Brien et al., 2003); and culture of neonatal testis tissue fragments (Sato et al., 2011a, 2011b). Translating these *in vitro* technologies to human cells for basic science research on gametogenesis will require appropriate oversight and review under existing mandates and/or committees for procuring and working with human tissues and cells. Using the human IVG-derived gametes for fertilization to create human embryos will require specialized scientific and ethics oversight as detailed below.

Starting with human ESCs and iPSCs, the initial steps of IVG to generate PGCLCs have been widely reported (Chen et al., 2017; Irie et al., 2015; Sasaki et al., 2015). In addition, human PGCLCs have the capacity to differentiate into human oogonia-like cells and oocytes (Yamashiro et al., 2018). However, the creation of ovarian follicles containing oocytes equivalent to those found in the adult human ovary remains to be achieved. Furthermore, the differentiation of GSCLCs or sperm from human cells has not been documented. IVG with immature human follicles isolated from the ovary (also called *in vitro* growth) before IVM is an active area of research. Safety concerns using IVG of follicles before IVM should also be considered as this is a critical window when imprints are re-established and meiosis resumes (Telfer, 2019). Together, these studies indicate that IVG with human cells is promising technology for restoring fertility. Yet a broad societal discussion is still needed, particularly when beginning with human pluripotent stem cells.

Based on this background, the committee recommended that basic research on human IVG without experiments designed to fertilize the resulting gametes should be permissible as a category 1B research activity. Although expected to be rare, it is theoretically feasible that under some circumstances parthenogenetic embryos may spontaneously develop from gametes produced by IVG. Given this, it is suggested that investigators report the creation of IVG-derived parthenogenetic embryos to a specialized scientific and ethics oversight process. This will enable review of the project and a determination as to whether future research should remain category 1B or comprehensively reviewed under category 2.

For scientists engaging in IVG with 46, XX or 46, XY cells where the research project involves IVM and fertilizing gametes to create human IVG-derived embryos, this research is permissible provided that embryos are maintained *in vitro* only. Such research must be reviewed under category 2 by a specialized scientific and ethics oversight process. Examples of permissible experiments could include the study of IVG-derived embryos up to 14 days post fertilization or formation of the primitive streak, whichever occurs first, or the derivation of cell lines from IVG-derived embryos. Experiments designed to transfer an IVG-derived human embryo into the uterus of a non-human animal host should not be pursued at all. Such experiments would be considered a category 3B activity because of broad international consensus that such experiments lack a compelling scientific rationale and are widely considered to be unethical. Similarly, research into which animal chimeras incorporating human cells with the potential to form human gametes are bred to each other is also considered a category 3B activity.

Finally, there is no compelling scientific evidence that IVG is currently safe for use in human reproduction, particularly when starting with hESCs, iPSCs, or iPSC derivatives, including PGCLCs, oogonia- or oocyte-like cells, or GSCLCs. This is because of unresolved issues related to epigenetic and genetic abnormalities of the resulting gametes, particularly given that the mouse oocytes and mouse GSCLCs derived from stem cells are reported to be of lower quality than their *in vivo* counterparts (Hikabe et al., 2016; Ishikura et al., 2016). Therefore, it was recommended that IVG for human reproductive purposes be categorized as a currently prohibited research activity until safety and ethical issues are resolved (category 3A). It was recognized that this technology will have the potential for use in human reproduction once safety and efficacy is proven, with the most promising approach likely to be IVG from immature follicles collected and frozen as part of fertility preservation before cancer treatment or sterility-inducing bone marrow transplants (Medicine, 2019). Furthermore, IVG and IVM to create sperm from pre-pubertal tissue may not be far behind.

In summary, the 2021 ISSCR Guidelines were updated to include a new regulatory and ethical framework for the oversight of IVG research and the creation of human embryos after IVG. This new framework recognizes the importance of IVG to generate basic science knowledge on the cell and molecular regulation of human germ cell development and human reproduction. In addition, by creating category 3A, the updated guidelines leave open the possibly that IVG could be used in the future to treat infertility if proven safe and remaining ethical issues are resolved.

### Conclusion

While recognizing that science moves faster than any set of guidelines and regulations can possibly respond, we hope that the ISSCR 2021 guidelines (ISSCR.org/guidelines) on human embryo research are flexible and far-sighted enough to provide the international community with some thoughtful guidance in considering and reviewing new and important areas of research.
